# Ten simple rules for conducting a mendelian randomization study

**DOI:** 10.1371/journal.pcbi.1009238

**Published:** 2021-08-12

**Authors:** Sarah A. Gagliano Taliun, David M. Evans

**Affiliations:** 1 Faculté de Médecine, Université de Montréal, Québec, Canada; 2 Montréal Heart Institute, Montréal, Québec, Canada; 3 Institute for Molecular Bioscience, University of Queensland, Brisbane, Queensland, Australia; 4 MRC Integrative Epidemiology Unit, University of Bristol, Oakfield House, Bristol, United Kingdom; 5 University of Queensland Diamantina Institute, Translational Research Institute, University of Queensland, Brisbane, Queensland, Australia; Carnegie Mellon University, UNITED STATES

## Introduction

**Fig 1:**
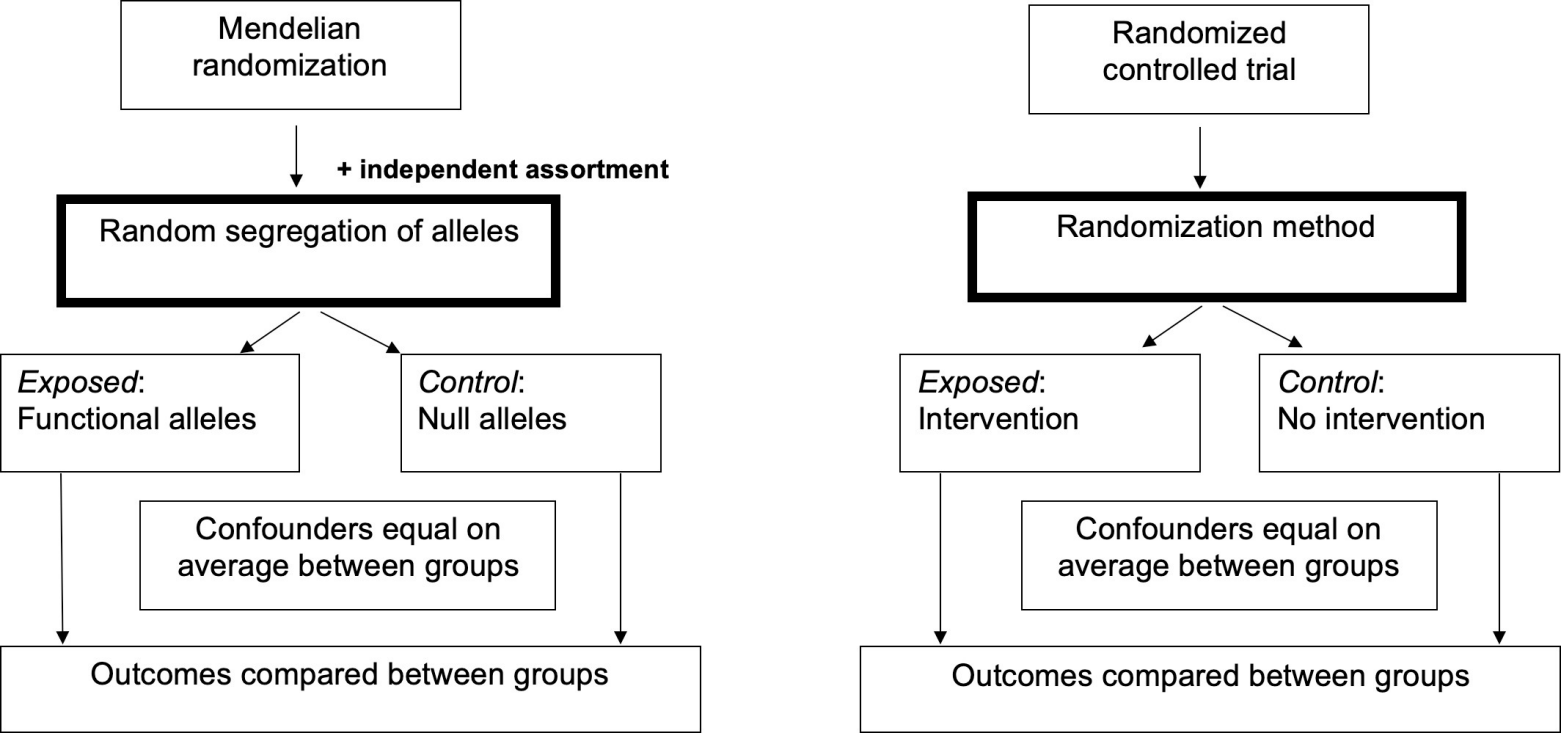
Similarities between the MR study design and a randomized controlled trial. MR, mendelian randomization.

Mendelian randomization (MR) is an epidemiological technique for estimating causal relationships using observational data, which has become very popular in recent years following publication of a seminal article by Smith and Ebrahim in 2003 [1]. MR is a specific form of “instrumental variables” (IV) analysis (the latter being first invented by Phillip and Sewall Wright in the 1920s [2]) that uses genetic variants to proxy a modifiable variable (which we term the “exposure” variable here) in order to estimate the causal relationship between the exposure and an outcome of interest. To understand how this causal inference technique works, it is useful to think of MR as similar to a “natural” randomized controlled trial [3] where individuals are randomly assigned to groups based on the alleles that they inherit from their parents (Fig 1). MR takes advantage of Mendel’s laws of segregation and independent assortment, which state that offspring inherit alleles randomly from their parents and randomly with respect to other genes in the genome (with certain exceptions [1]). Therefore, genetic variants that are related to an exposure of interest can be used to proxy the part of the exposure variable that is independent of possible confounding influences from the environment and other traits. Providing several assumptions are satisfied (see below), and the principle of gene–environment equivalence (i.e., perturbing the exposure genetically has the same effect as perturbing the exposure by other means), statistical association between the genetic variant and the outcome is indicative of a causal relationship between the exposure and the outcome and can be used to estimate the magnitude of the causal relationship using IV methods. Although originally developed as a way to estimate causal relationships between modifiable environmental exposures and medically relevant outcomes, in recent years, MR has been utilized in many other situations including studies of molecular biomarkers, in pharmacogenetics, in the social sciences, and in other discplines that use observational frameworks [4,5].

Given the growing number of MR studies in the literature and the increasing amount of publicly available genome-wide association study (GWAS) datasets and variant–trait association summary statistics, which make such studies feasible, we describe 10 simple rules for conducting an MR study. Our aim is not to provide a comprehensive and detailed overview of MR (which can be found elsewhere [4–6]), but rather to present a starting place for researchers to prepare to conduct and to begin to critically evaluate existing MR studies.

## Rule 1: Have a clear research question

Specify your relationship of interest; that is to say, does having trait A (exposure) or having a particular level/dose of trait A cause trait B (outcome)? “Trait” should be interpreted broadly and could refer to, for example, a disease, an environmental exposure, a molecular biomarker, and/or a quantitative trait. Often, the exposure is modifiable (alcohol consumption or vitamin D levels are 2 examples), so that there is a potential opportunity to intervene on the variable if the MR analyses provide evidence supporting a causal relationship between the exposure and the outcome. Nevertheless, investigating factors that are not easily modifiable (such as adult height or birth weight) in an MR framework can also be informative from a mechanistic perspective. The interrogation of causality by MR does not necessarily involve a single exposure and outcome pair. You could, for example, conduct an MR–phenome-wide association study [7] looking at potential causal relationships between a single exposure and multiple outcomes, or, alternatively, between multiple exposures and a single outcome. Regardless of the choice of exposure(s) and outcome(s), your underlying research question must be clear.

## Rule 2: Keep in mind the core IV assumptions

There are 3 core assumptions genetic variants must satisfy in order to be considered IVs for testing hypotheses about whether an exposure is causally related to an outcome of interest. The first is that the genetic variants used to proxy for the exposure are robustly associated with the exposure. The second assumption is that there is no confounding (measured or unmeasured) of the genetic variants with the outcome. The third assumption is that the variants potentially influence the outcome only through the exposure. Only the first of these assumptions can be proven definitively. That is to say, you can obtain statistical evidence that your genetic variants are related to the exposure and compute a measure (typically, the F-statistic from a regression of the exposure on the variant is used) of the strength of this association [8]. The more variance the genetic variant explains in the exposure and the larger your sample size, the more powerful your analysis and the more accurate and precise your estimate of the causal effect of exposure on outcome. For the remaining assumptions, sensitivity analyses should be performed, if possible, to assess whether the assumptions are likely to have been violated. For example, different genetic variants exhibiting differences in the magnitude of the estimated causal effect suggest (i) the presence of horizontal pleiotropy (where a variant affects multiple phenotypes); and (ii) violation of the third IV assumption. In the case of the second IV assumption, Mendel’s laws of segregation and independent assortment strongly suggest that genetic variants should be unrelated to environmental and genetic confounding variables, respectively. Empirical tests of the relationship between genetic variants and known confounders of the exposure–outcome relationship can increase confidence in the validity of this assumption but are not definitive in that other unmeasured confounders of the exposure–outcome relationship could still be associated with the genetic variant. Additionally, other processes can generate spurious associations between the genetic variant and the outcome including population stratification, selection bias, and dynastic effects [9]. Investigators should be particularly cognizant of the potential for population stratification to reintroduce confounding into the MR analysis and take actionable steps to control for this possibility, such as including ancestry informative principal components in the statistical model.

While the above 3 core assumptions are sufficient for testing whether an exposure causes the outcome, in order to obtain accurate point estimates of the causal effect, further (strong) assumptions regarding the form of the relationship between the genetic variant, exposure, and outcome also need to be made (e.g., linearity) [10].

## Rule 3: Be attentive when selecting genetic variants to be used as instruments

Decreasing genotyping costs, the emergence of large-scale biobanks and GWAS meta-analytic consortia, and the widespread availability of variant–trait association summary statistics and databases in the public domain, such as MR-Base [11], have facilitated the identification and utilization of genetic instruments for MR studies. There are no set rules for selecting the “best” set of genetic instruments for an MR study. For example, a well-powered MR analysis using a single variant with a well-understood mechanism of action (and unlikely to involve horizontal pleiotropy) may be a superior strategy to performing an MR study as opposed to using as many genome-wide significant variants as possible to proxy the exposure [12]. Decisions related to genetic instrument selection are made on a case-by-case basis, but guidelines have been developed to assist in this process [13]. Important considerations include strength of the variant–exposure association (the more robust the better), variant independence (genetic variants should not be in linkage disequilibrium unless this correlation is explicitly modeled in the analysis), and the likelihood of horizontal pleiotropy (which may be a rationale for variant exclusion). Selecting genetic variants that are appropriate for your sample is also key; in particular, it is important to be aware of any variants that exert ancestry-, sex-, or age-dependent effects.

## Rule 4: Consider the possibility of reverse causality

Do the genetic variants exhibit their primary association with the exposure variable as opposed to the outcome variable? For example, if variable A has a large causal effect on variable B, then genetic variants primarily associated with variable A will reach genome-wide significance in a GWAS of variable B given a large enough sample. These variants could be erroneously used as instruments for estimating the causal effect of variable B on variable A when in fact their primary association is with variable A. The use of such variants would bias the results of MR analyses of the causal effect of variable B on variable A and potentially provide spurious evidence of reverse causality due to misspecification of the primary trait. Steiger filtering [14] can be used to identify a set of genetic variants that have their primary association with the exposure of interest. If bidirectional causal relationships are a possibility (i.e., variable A causes variable B, and variable B causes variable A), then consider using “reciprocal MR” in which exposure and outcome are instrumented and MR performed in both directions [15].

## Rule 5: Understand the pros and cons of using one- versus two-sample MR

Perform one- or two-sample MR using one of the many well-documented software packages that are freely available (e.g., the “two-sample MR package” in the R statistics software) or the MR-Base web utility that uses the MRC IEU OpenGWAS data infrastructure of harmonized GWAS summary datasets and metadata [11,16]. One-sample MR has the advantage that it is possible to confirm that genetic markers used in the analysis are independent of known confounding variables and also permits many specialized types of MR analysis (e.g., gene by environment MR [17], factorial MR [18], and nonlinear MR [19]). Potential disadvantages are that large samples may be difficult to obtain, which lowers power, and that any bias from weak instruments (i.e., genetic markers that are not robustly related to the exposure in the sample under study) will be in the direction of the observational association [8]. Two-sample MR (i.e., obtaining variant–exposure and variant–outcome effect sizes from 2 different datasets) is often advantageous in terms of statistical power, in that publicly available summary results data from large genome-wide association consortia can be used inexpensively, easily, and efficiently. This approach can boost sample size and facilitate the analysis of expensive/hard to measure exposures or outcomes. However, it assumes that the different exposure and outcome datasets are ancestrally homogeneous and that the same causal process operates in both datasets. Any bias from weak instruments tends to be toward the null [4,20]. If performing two-sample MR, ensure that the effect of the variant on the exposure variable and the effect of the variant on the outcome variable correspond to the same allele. Be careful if using pallindromic variants (i.e., A/T or C/G variants), so that variant–exposure and variant–outcome effect estimates correspond to the same strand. If using several independent variants, causal effect estimates can be combined by weighting them by the inverse of their variance (i.e., termed the “inverse variance–weighted (IVW) MR” method).

## Rule 6: Visualize results

Graphical visualization can be useful for checking the validity of MR assumptions. Forest plots, which display causal effect estimates across the different genetic variants, can be useful in terms of identifying outliers and potential pleiotropic variants. Funnel plots, which graph variant instrument strength (y-axis) against causal effect estimate, can be useful in identifying the presence of directional pleiotropy in the data.

## Rule 7: Run sensitivity analyses to increase confidence in the validity of the results

While IVW MR is the most statistically powerful approach to combine/meta-analyze causal effect estimates [21], it assumes the complete absence of horizontal pleiotropy. Therefore, perform tests of heterogeniety to investigate whether estimates of the causal effect differ across the various genetic variants [22]. Different causal effect estimates suggest the presence of horizontal pleiotropy and can flag outlying variants for further investigation. Perform sensitivity analyses that relax the strict assumption of no horizontal pleiotropy. These different methods include, but are not limited to, random effects MR, the MR modal-based estimator [23], weighted median MR [24], MR–Egger regression [25], MR–robust adjusted profile score (RAPS) [26], and simulation-based heterogeneity and outlier tests (e.g., MR–pleiotropy residual sum and outlier (PRESSO)) [27]. Also consider assessing potential biases due to measurement error in the variant–exposure associations [28], sample overlap [29], and selection bias [30]. Consistent causal effect estimates across the different methods improve confidence in the validity of the MR results. In certain cases, it may be possible to utilize an informative gene by environment interaction to inform on the presence or absence of horizontal genetic pleiotropy. For example, in MR studies of the relationship between alcohol consumption and disease outcomes, an association between genetic variants proxying number of units of alcohol consumed per day and disease status should not be present in the subpopulation of individuals who do not consume alcohol. Indeed, the existence of such associations may indicate the presence of horizontal pleiotropy.

## Rule 8: Document code and ensure reproducibility

Replication is essential for advancing science, and code transparency in computational research is a key step in facilitating reproducibility. There are papers to help with fostering reproducible computational research, including from the Ten Simple Rules series of *PLOS Computational Biology* [31]. With this premise in mind, code should be clear, concise, and well documented in a manner that allows others to replicate your results. Using an online open-source code collaboration tool, such as GitHub (https://github.com), and getting your code independently tested are useful ways to share code and verify reproducibility.

Guidelines for reporting MR studies have been proposed [32]. As well as sharing code, it is helpful to document how the datasets have been constructed, such as the characteristics of the participants in the GWAS studies (especially in cases where sharing of individual-level datasets is not possible), and to present detailed summary results data (effect alleles, strand, effect sizes, allele frequencies, *p*-values, etc.) for the individual genetic variants used to proxy for the exposure.

## Rule 9: Carefully interpret results and acknowledge limitations

The critical appraisal checklist available in a review paper on interpreting MR studies offers concrete guidance for the interpretation of results [33]. Of note, an essential point to consider when interpreting such studies is whether gene–environment equivalence is reasonable; i.e., do changes caused by genotypes have the same downstream effects as if they were caused by modifiable exposures? Additionally, results from MR cannot necessarily be generalized to individuals who differ from those from which the effect sizes were derived, such as individuals from different ancestries, environments, sexes, and ages. The nontransferability of results is one reason why it is crucial to provide detailed information on the characteristics of the datasets used in the analysis, as noted in the previous rule.

## Rule 10: Disseminate findings to the research community

Now it is time to formally share the results from your efforts with colleagues and the broader research community through a scientific publication and/or a conference presentation. There is a helpful advice provided in earlier articles of this Ten Simple Rules series of *PLOS Computational Biology* for disseminating research through written and oral communication [34,35].

## Conclusions

MR uses genetic variant–trait associations to estimate the causal effect of an exposure variable on an outcome. Originally developed to estimate causal relationships between modifiable environmental exposures and medically relevant outcomes, the scope of the MR paradigm has widened to include applications in fields as diverse as molecular biology, pharmacology, and the social sciences. When conducted appropriately and its results triangulated with substantive knowledge and results using other research methodologies, MR can be a powerful tool for informing causality.
